# Expression of genes and enzymes involved in ovarian steroidogenesis in relation to human follicular development

**DOI:** 10.3389/fendo.2023.1268248

**Published:** 2023-10-26

**Authors:** Mengxue Zheng, Claus Yding Andersen, Frida Roikjer Rasmussen, Jesús Cadenas, Søren Tvorup Christensen, Linn Salto Mamsen

**Affiliations:** ^1^ Laboratory of Reproductive Biology, The Juliane Marie Centre for Women, Children and Reproduction, Copenhagen University Hospital, Copenhagen, Denmark; ^2^ Department of Clinical Medicine, Faculty of Health and Medical Science, University of Copenhagen, Copenhagen, Denmark; ^3^ Department of Cell Biology and Physiology, University of Copenhagen, Copenhagen, Denmark

**Keywords:** human ovarian steroidogenesis, progesterone, 17-hydroxy -progesterone, oestrogen, theca cells, granulosa cells

## Abstract

**Introduction:**

Granulosa cells (GCs) and theca cells (TCs) play a pivotal role in human ovarian steroidogenesis, facilitating the conversion of cholesterol into sex steroids that regulate normal reproductive function. This study aims to explore the expression patterns of key enzymes that govern human ovarian steroidogenesis throughout follicle development, employing both genomic and immunological methodologies.

**Methods:**

Follicles and GCs obtained from women undergoing ovarian tissue cryopreservation (OTC) and *in vitro* fertilisation treatment were utilized. Gene expression data were obtained from a Chinese study using RNA sequencing and from microarray data generated in our laboratory to comprehensively analyse gene expression profiles across distinct stages of follicular development. To corroborate the localisation of key enzymes within GCs and TCs, immunohistochemistry analyses utilizing colourimetric and fluorescent techniques were conducted.

**Results:**

Steroidogenesis-related enzymes displayed low gene expression levels during early follicle development. However, a notable upregulation of *HSD3B2* was observed in GCs as follicles progressed to the antral/preovulatory stage, confirmed consistently using both microarray and RNA sequencing methodologies. Furthermore, immunohistochemical analyses effectively demonstrated that HSD3B2 were not only expressed in GCs, but co-localised with CYP17A1 within a specific subset of TCs surrounding human small antral follicles. Contributing to an enhanced progesterone production during the second half of the follicular phase was a significant upregulation of *CYB5A* in both microarray and RNA-seq datasets as follicles transition from the antral stage to the pre-ovulatory stage. Moreover, an augmented expression of *DHCR24* and *LDLR* in both types of data, along with *HMGCR* expression expression in the microarray data, indicates increased substrate availability for ovarian steroidogenesis.

**Discussion:**

This study confirms and extends that GCs gradually augment expression of HSD3B2 thereby enhancing their capacity for progesterone synthesis as follicles reach the size of selection at around 10 mm in diameter. This is supported by the expression *CYB5A* and possibly augmented availability of steroid precursors. A subset of TCs exhibit concurrent expression of CYP17A1 and HSD3B2, collectively contributing to the synthesis of 17-hydroxyprogesterone. These data significantly enhance our understanding of the dynamic regulation of progesterone throughout the process of follicular development.

## 1 Introduction

**Figure 1:**
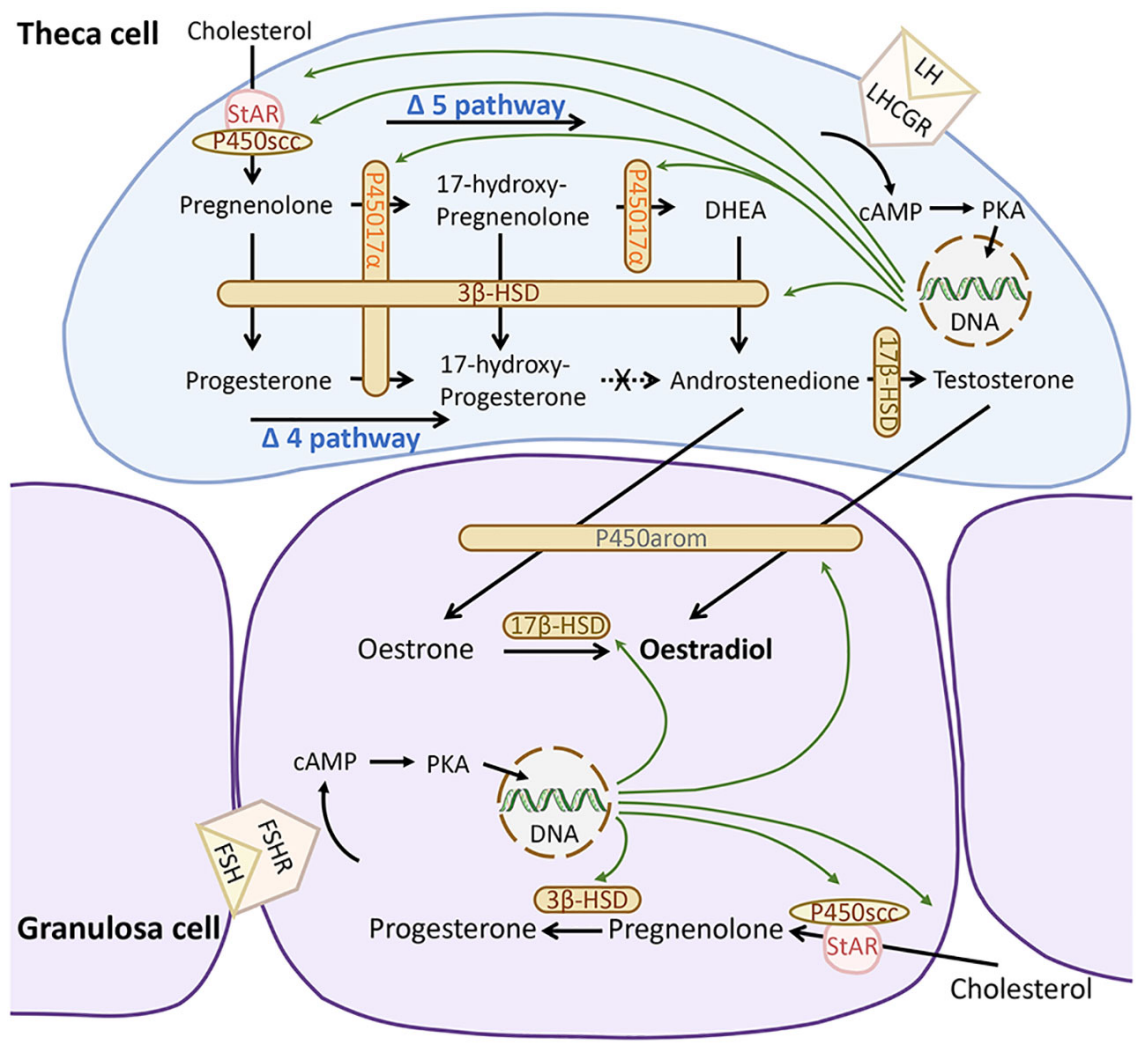
Human ovarian steroidogenesis. During the follicular phase of the menstrual cycle, the theca cells produce 17- hydroxy-progesterone and androgens, while granulosa cells produce oestrogens and progesterone. Sex steroid biosynthesis proceeds via either the Δ4 or Δ5 pathway. Via the Δ4 pathway pregnenolone is converted to progesterone and then 17-hydroxy-progesterone. In humans, the conversion of 17-hydroxy-progesterone to androstenedione is limited. Through the Δ5 pathway, pregnenolone is synthesized via 17-hydroxy-pregnelonone, DHEA into androstenedione, which continues to be converted and aromatized into oestrogens (oestrone or oestradiol). FSH, follicle-stimulating hormone; FSHR, follicle-stimulating hormone receptors; LH, luteinising hormone; LHCGR; LHR, luteinising hormone/choriogonadotropin receptor; HSD, Hydroxysteroid dehydrogenase; DHEA, dehydroepiandrosterone; cAMP, cyclic adenosine monophosphate; PKA, protein kinase A. Produced with PowerPoint.

Human ovarian steroidogenesis almost exclusively occurs in follicles, where granulosa cells (GCs) and theca cells (TCs) play pivotal roles in converting cholesterol into sex steroids essential for normal reproductive function (1, 2). The final product of this process is typically oestradiol, synthesized through a sequence of enzymatic reactions involving both TCs and GCs (**Figure 1**). In women, the Δ5 pathway primarily governs oestradiol synthesis, as the enzyme 17α-hydroxylase/17,20 lyase (CYP17A1) exhibits limited activity in converting 17-hydroxyprogesterone (17-OH-P_4_) to androstenedione (1, 2). Consequently, steroids produced via the Δ4 pathway, such as progesterone (P_4_) and 17-OH-P_4_, serve as terminal products due to the unidirectional action of hydroxy-delta-5-steroid dehydrogenase, 3 beta- and steroid delta-isomerase 2 (HSD3B2). Specifically expressed in TCs, CYP17A1 converts P_4_ to 17-OH-P_4_, which remains unmetabolized. Conversely, GCs lack CYP17A1 expression, resulting in P_4_ serving as a terminal product within this cell population.

The synthesis of sex steroids is under the control of gonadotropins, notably follicle-stimulating hormone (FSH) and luteinising hormone (LH). While luteinizing hormone/chorionic gonadotropin receptors (LHCGR) are constitutively expressed on TCs and are concurrently expressed on GCs when follicular diameter exceeds 10 mm (3), follicle-stimulating hormone receptors (FSHR) are exclusively present on GCs (**Figure 1**).

Steroidogenic activity within follicles intensifies with developmental progression. During the latter half of the follicular phase, the preovulatory follicle experiences peak production and secretion of sex steroids especially P_4_ and oestradiol. In a natural menstrual cycle, the chosen preovulatory follicle generates over 90% of the circulating oestrogen. Moreover, P_4_ secretion increases as follicular diameter surpasses 10 mm. Notably, it has been proposed that P_4_ accumulates within follicular fluid until shortly before ovulation (4). TCs produce concentrations of 17-OH-P_4_ that exceed circulating P_4_ levels during the follicular phase (5).

However, the extent to which TCs also secrete P_4_ remains uncertain. The TC layer consists of various sub-types (6), and it remains unclear if a specific sub-type expresses both HSD3B2 and CYP17A1, prerequisites for synthesizing 17-OH-P_4_ within a single cell. Furthermore, the activity of the key enzymes in ovarian steroidogenesis is also affected by a number of co-factors like cytochrome b5 (Cyt-b5), cytochrome p450 oxidoreductase (POR) and aldo-keto reductase family 1 member C3 (AKR1C3), also known as 17β-hydroxysteroid dehydrogenase type 5 (HSD17B5), which may catabolize P_4_ and oestradiol (7).

Also, the substrate availability of cholesterol for further metabolism may affect the synthesis of ovarian sex steroids, including expression of HMG-CoA reductase (3-hydroxy-3-methylglutaryl-coenzyme A reductase, HMGCR), which is the rate-controlling enzyme of the mevalonate pathway which leads to cholesterol production and 24-dehydrocholesterol reductase (DHCR24) which is important for cholesterol synthesis. The low density lipoprotein receptor (LDLR) is central for the uptake of cholesterol from circulation.

This study’s primary objective is to elucidate the expressions of key enzymes, including CYP17A1 and HSD3B2, which regulate human ovarian steroidogenesis during follicle development. The investigation aims to delineate temporal expression patterns of these enzymes, employing whole-genome microarray, RNA sequencing data, and immunohistochemistry techniques.

## 2 Materials and methods

### 2.1 Samples for microarray data

Follicles, including those from preantral, small antral, and preovulatory stages, were procured from women aged 23 to 36 for messenger ribonucleic acid (mRNA) microarray analysis. These datasets have been previously documented in published studies (8–11), and their summarized details are presented in **Table 1**.

**Table 1:** Overview of the human follicles/granulosa cells samples for microarray analysis.

Stage	Materials	Sample count	Datasets	Previous papers
**Preantral Follicle**	Pooled preantral follicles 40 to 200 µm in diameter	17	E-MEXP-3783	Kristensen et al., 2015 (9)
**Small Antral Follicle**	GCs isolated from antral follicles 4 to 6 mm in diameter	3	E-MTAB-2862	Petersen et al., 2015 (11)
**Preovulatory follicle (Pre-OI)**	GCs isolated from antral follicles 14 to 17mm in diameter 13-61 h before hCG treatment for ovulation induction	9	E-MTAB-2203	Wissing et al., 2014 (10)
**Mural granulosa cells (post-OI)**	Mural GCs isolated from preovulatory follicles after 34/36-h hCG or GnRHa administration for ovulation induction	25	E-MTAB-2203 E-MTAB-1670	Wissing et al., 2014 (10) Borgbo et al., 2013 (8)
**Cumulus cells** **(post-OI)**	Cumulus cells isolated from preovulatory follicles after 34-h hCG or GnRHa administration for ovulation induction	14	E-MTAB-1670	Borgbo et al., 2013 (8)

OI, ovulation induction; GCs, granulosa cells; hCG, human chorionic gonadotropin; GnRHa, gonadotropin-releasing hormone agonist.

Preantral and small antral follicles were harvested from women undergoing fertility preservation who had one ovary surgically excised (9, 11). For fertility preservation purposes, only the ovarian cortex containing primordial follicles was utilized, while the medullary tissue was either discarded or allocated for research, subject to patient consent (12).

In addition, small antral follicles were procured from the medulla tissue of two patients undergoing fertility preservation for subsequent immunohistochemical (IHC) and immunofluorescence (IF) analysis. These patients, aged 30 and 29 years, had been diagnosed with breast cancer and cervical cancer, respectively.

Granulosa cells were collected from preovulatory follicles both before induction of final maturation (pre-OI) and at oocyte pickup (post-OI). These cells were obtained from women undergoing ovarian stimulation for *in vitro* fertilization (IVF) treatment. Paired samples of GCs were acquired from nine patients: one sample pre-OI and another at oocyte pickup (OPU). These samples were subjected to microarray analysis; the mean age of the patients was 27.9 ± 3.4 (SD) (10).

Furthermore, mural GCs and cumulus cells from preovulatory follicles at the time of OPU were obtained from women undergoing IVF or intracytoplasmic sperm injection for microarray analysis (8). These patients received either 5000 IU hCG (pregnyl, MSD) or 0.5 mg GnRHa (Suprefact, Sanofi-Aventis) for final maturation of follicles.

### 2.2 Ethical approval

The utilization of surplus ovarian tissue, encompassing follicles and granulosa cells, obtained in conjunction with ovarian tissue cryopreservation (OTC), received approval from the Scientific Ethical Committee for the Capital Region (Approval No. H-2-2011-044).The collection and use of GCs from both pre- and post-OI follicles, acquired during IVF treatment, were conducted in compliance with the Helsinki Declaration II and were approved by the Danish Scientific Ethical Committee (Approval No. SJ-156). The utilization of paired mural granulosa cells and cumulus cells from preovulatory follicles at OPU in connection with IVF treatment was approved by the Danish Ethical Committee (Approval No. VN2004/61). Informed consent was obtained from all participants prior to their inclusion.

### 2.3 Processing of surplus ovarian tissue from OTC

Preantral follicles, ranging in diameter from 40 µm to 200 μm, displaying an intact membrane with granulosa cells and an oocyte, were isolated from the medullary tissue, following established protocols (9, 13). These follicles were rapidly frozen in liquid nitrogen or lysed in RNA lysis buffer and subsequently stored at -80 °C until RNA extraction.

Small antral follicles, measuring 4 to 6 mm in diameter, designated for microarray analysis, were aspirated using a 26 G needle attached to a 1 mL syringe from ovaries acquired for fertility preservation intended for OTC. Following aspiration, the follicle fluid (FF) and GCs were separated through centrifugation at 300g for 2 minutes. The isolated GCs were then subjected to two washes with PBS and preserved at -80 °C until further analysis, as previously described (14).

Additionally, small antral follicles, ranging from 0.5 to 6 mm in diameter and isolated from the medullary tissue for histological analysis, were either fixed in Bouin’s solution for immunohistochemical analysis or in formalin for immunofluorescence analysis. Subsequent, they underwent a series of washes in graded ethanol, were embedded in paraffin, and sectioned into 5 μm sections.

### 2.4 Processing of GCs from women undergoing IVF or ICSI

The paired pre- and post-OI follicles were punctured using transvaginal ultrasound guidance at 13 hours (N=4), 37 hours (N=4) and 61 hours (N=1) prior to hCG administration (pre-OI) and at post-OI (10). The isolation of GCs was described previously (15). Mural GCs and cumulus cells post-OI were obtained 34 hours after hCG or gonadotropin-releasing hormone agonist (GnRHa) administration, as previously described (8).

### 2.5 Microarray data

All microarray data sets were conducted at the same core facility (the Microarray Center at Rigshospitalet, Copenhagen) and used the Affymetrix Human Gene ST v1.0 GeneChip array (Affymetrix, Santa Clara, California, USA). The microarray data were validated using RT-PCR and the results are available in previous publications (8–11).

Cell intensity files, commonly known as CEL files, were generated using the GeneChip Command Console software (AGCC, Affymetrix). Subsequently, these CEL files were imported into the R software environment (version 4.2.2) (16, 17) for further analysis. To enhance the quality and reliability of the microarray data, we employed Frozen Robust Multiarray Analysis (fRMA, version 3.16) (18) for microarray preprocessing. This method not only combined data from different batches to mitigate technical variability but also normalized the data to eliminate batch-specific differences, thereby enhancing data reproducibility and comparability. Microarray gene expression data were presented as log_2_-transformed expression levels.

We excluded samples that were contaminated with leukocytes. The presence of leukocyte contamination was determined by assessing the expression of the leukocyte-specific marker cluster of differentiation 45 (*CD45*), also known as the protein tyrosine phosphatase receptor type C (*PTPRC*) gene. Two samples displaying elevated *PTPRC* expression were consequently excluded from the study. However, the remaining samples exhibited low *PTPRC* expression, and these expression levels did not exhibit statistically significant differences between the samples (see **Supplementary Table S1**, **Supplementary Figure S1**).

**Figure 2:**
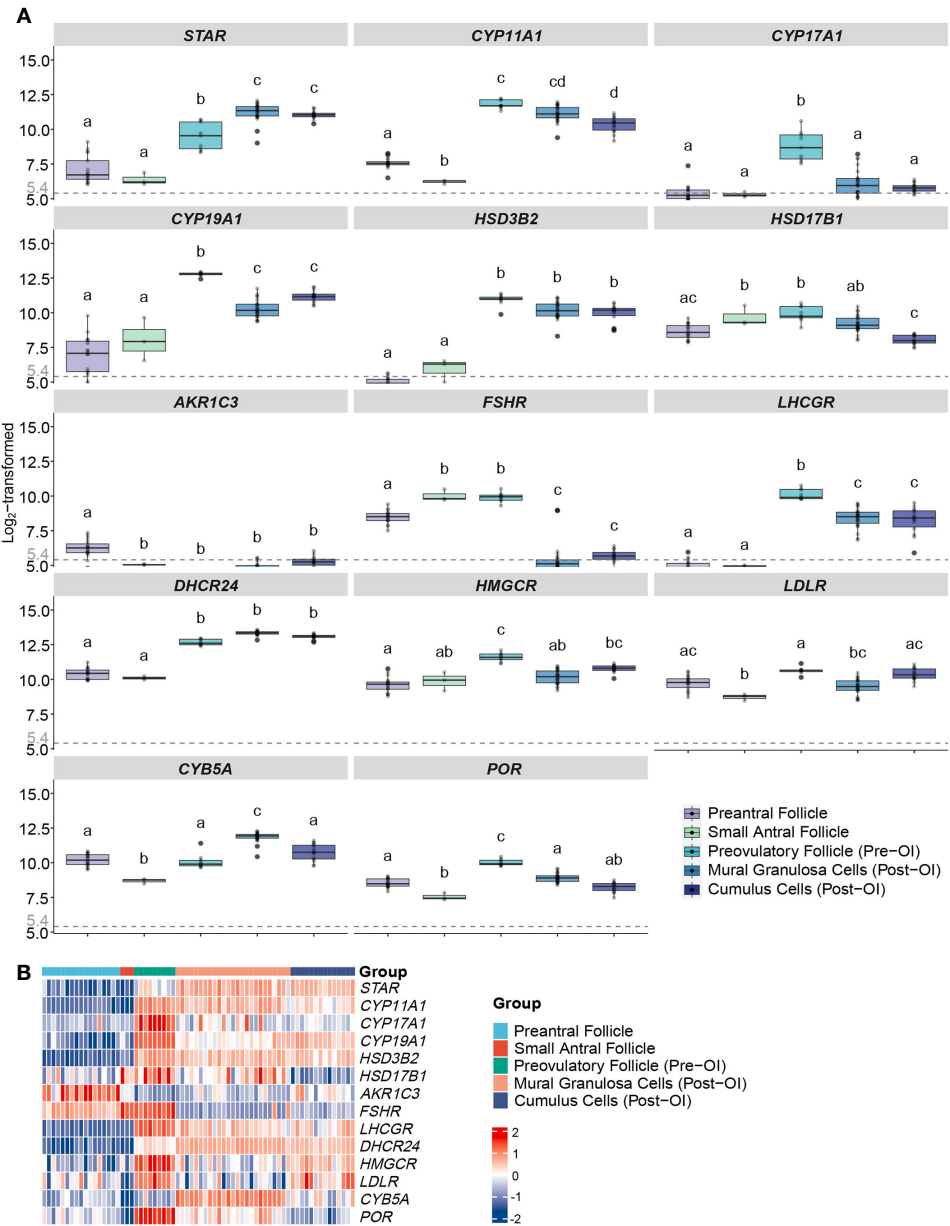
Expression profiles of steroidogenesis-related genes in human follicles/GCs at different follicular stages from microarrays. **(A)** Boxplots of genes related to ovarian steroidogenesis expressed in human follicles/GCs at different follicular stages. Log_2_-transformed expression levels of each gene from the microarray gene expression datasets are displayed. The x-axes show different groups of follicles, which are preantral follicles from 40 to 200 µm in diameter, GCs from 5 to 10 mm small antral follicle, GCs from follicles before ovulation induction larger than 16 mm, mural GCs after ovulation induction, cumulus cells after ovulation induction. Different letters between follicle/GCs groups indicate statistical significance. Values below 5.4 are regarded as background noise. **(B)** Heatmap of genes expressed in human follicles/GCs at different follicular stages. The expression level of each gene between different samples is presented by the intensity of the colour. OI, ovulation induction; GCs, granulosa cells.

To gauge the level of gene expression noise in the arrays, we utilized the expression of two Y-chromosome-located genes, namely testis-specific protein Y-linked 1 (*TSPY1*) and deleted in azoospermia 4 (*DAZ4*). The log_2_ expression values of *TSPY1* and *DAZ4* ranged from 3.2 to 5.4, as documented in **Table 2** and **Figure 2A**. Importantly, no statistically significant differences were observed between the groups, as indicated by *q* values exceeding 0.05, and the absolute log_2_ fold changes remained less than 1. In this study, we considered log_2_ gene expression levels below 5.4 as indicative of noise.

**Table 2:** Expression levels of human follicle/granulosa cell selection genes in microarray analysis.

Log_2_-transformed	Preantral Follicle	Small Antral Follicle	Preovulatory Follicle (Pre-OI)	Mural Granulosa Cells (Post-OI)	Cumulus Cells (Post-OI)
	(N= 17)	(N= 3)	(N= 9)	(N= 25)	(N= 14)
*STAR*					
Mean (SD)	7.1 (1.0)	6.4 (0.5)	9.5 (1.0)	11.2 (0.7)	11.0 (0.3)
Median [Min, Max]	6.7 [6.0, 9.1]	6.2 [6.1, 6.9]	9.5 [8.3, 10.7]	11.3 [9.0, 12.1]	11.0 [10.4, 11.5]
*CYP11A1*					
Mean (SD)	7.6 (0.4)	6.2 (0.1)	11.8 (0.3)	11.1 (0.6)	10.3 (0.6)
Median [Min, Max]	7.5 [6.5, 8.3]	6.3 [6.1, 6.3]	11.7 [11.3, 12.2]	11.1 [9.4, 12.0]	10.5 [9.2, 11.2]
*CYP17A1*					
Mean (SD)	5.4 (0.6)	5.3 (0.2)	8.8 (1.1)	6.1 (1.0)	5.8 (0.4)
Median [Min, Max]	5.2 [4.9, 7.4]	5.3 [5.1, 5.5]	8.7 [7.5, 10.6]	5.9 [4.8, 8.2]	5.8 [5.3, 6.4]
*CYP19A1*					
Mean (SD)	7.0 (1.3)	8.0 (1.6)	12.8 (0.2)	10.3 (0.6)	11.2 (0.4)
Median [Min, Max]	7.1 [4.9, 9.8]	7.9 [6.5, 9.7]	12.8 [12.4, 12.9]	10.2 [9.4, 11.7]	11.2 [10.5, 11.9]
*HSD3B2*					
Mean (SD)	5.0 (0.4)	5.9 (0.8)	10.9 (0.4)	10.2 (0.6)	9.9 (0.7)
Median [Min, Max]	4.9 [4.3, 5.7]	6.3 [5.0, 6.5]	11.0 [9.9, 11.4]	10.1 [8.3, 11.1]	10.2 [8.7, 10.7]
*HSD17B1*					
Mean (SD)	8.6 (0.6)	9.7 (0.7)	9.9 (0.6)	9.2 (0.6)	8.0 (0.4)
Median [Min, Max]	8.6 [7.8, 9.6]	9.3 [9.2, 10.5]	9.7 [8.9, 10.7]	9.1 [8.0, 10.4]	8.0 [7.5, 8.5]
*AKR1C3*					
Mean (SD)	6.2 (0.6)	5.1 (0.0)	4.4 (0.3)	4.8 (0.4)	5.2 (0.5)
Median [Min, Max]	6.3 [4.8, 7.4]	5.0 [5.0, 5.1]	4.4 [4.0, 4.7]	4.7 [4.3, 5.6]	5.3 [4.3, 6.1]
*FSHR*					
Mean (SD)	8.5 (0.5)	10.0 (0.4)	9.9 (0.3)	5.4 (1.1)	5.7 (0.4)
Median [Min, Max]	8.5 [7.5, 9.4]	9.8 [9.7, 10.5]	9.9 [9.3, 10.5]	5.1 [4.5, 9.0]	5.7 [4.7, 6.4]
*LHCGR*					
Mean (SD)	5.0 (0.43)	4.9 (0.1)	10.1 (0.4)	8.4 (0.70)	8.2 (1.0)
Median [Min, Max]	4.8 [4.3, 6.0]	4.9 [4.9, 5.0]	9.9 [9.8, 10.8]	8.5 [6.8, 9.5]	8.4 [5.9, 9.5]
*DHCR24*					
Mean (SD)	10.4 (0.4)	10.1 (0.1)	12.7 (0.2)	13.3 (0.1)	13.1 (0.2)
Median [Min, Max]	10.4 [9.9, 11.2]	10.1 [10.0, 10.2]	12.6 [12.4, 13.0]	13.4 [12.8, 13.6]	13.1 [12.7, 13.3]
*HMGCR*					
Mean (SD)	9.6 (0.6)	9.9 (0.7)	11.6 (0.3)	10.2 (0.5)	10.8 (0.3)
Median [Min, Max]	9.7 [8.8, 10.8]	9.9 [9.2, 10.5]	11.6 [11.1, 12.1]	10.2 [9.2, 11.0]	10.8 [10.1, 11.2]
*LDLR*					
Mean (SD)	9.7 (0.5)	8.7 (0.2)	10.6 (0.3)	9.4 (0.6)	10.4 (0.5)
Median [Min, Max]	9.8 [8.7, 11]	8.8 [8.5, 8.9]	10.7 [10.1, 11.1]	9.5 [8.5, 10.5]	10.3 [9.5, 11.1]
*CYB5A*					
Mean (SD)	10.2 (0.4)	8.7 (0.2)	10.1 (0.5)	11.8 (0.4)	10.7 (0.6)
Median [Min, Max]	10.2 [9.5, 10.8]	8.8 [8.5, 8.8]	9.9 [9.6, 11.4]	11.9 [10.4, 12.3]	10.8 [9.8, 11.6]
*POR*					
Mean (SD)	8.5 (0.3)	7.5 (0.3)	10.0 (0.2)	8.9 (0.3)	8.2 (0.4)
Median [Min, Max]	8.5 [7.9, 9.0]	7.4 [7.3, 7.8]	9.9 [9.8, 10]	8.9 [8.4, 9.6]	8.3 [7.5, 8.8]

OI, ovulation induction; N, sample number; SD, standard deviation; Min, minimum value; Max, maximum value.

### 2.6 RNA-seq data

Ovarian tissue from seven women were collected in connection with fertility preservation. None of the women received any kind of ovarian stimulation (19). RNA sequencing data (GSE107746) of human oocytes and corresponding GCs from 5 different follicular developmental stages were downloaded from the GEO database (https://www.ncbi.nlm.nih.gov/gds/). Only the data of GCs samples were extracted for the present study. Data normalisation and other pre-processing steps were skipped because the obtained data were already in the log_2_ of Fragments Per Kilobase of transcript per Million mapped reads (FPKM) plus one format. The data was first published by Zhang and co-workers (19).

### 2.7 Chromogenic immunohistochemistry

Chromogenic immunohistochemical analysis were conducted following established procedures (20). Briefly, tissue sections were de-paraffinized using tissue-clear solution (Sakura Finetek, Brøndby, Denmark, Cat. No.: 1466) and subsequently rehydrated through graded ethanol. Antigen retrieval was achieved using Tris-EGTA buffer (10 mM Tris, 0.5 mM EGTA, pH 9). To reduce endogenous peroxidase activity, 3% H_2_O_2_ was applied. Non-specific binding was blocked by incubating sections with 4% bovine serum albumin (BSA) and 5% species-specific normal serum (normal donkey serum, Abcam, Cat. No.: ab7475) before primary antibody application. Sections were incubated with primary antibodies overnight at 4°C in a humid chamber. Primary antibodies used included HSD17B1 (1:100, Abcam, Cambridge, UK, cat. no.: ab51045), CYP17A1 (1:200, Santa Cruz, Dallas, US, Cat. No.: SC-46084), and HSD3B2 (1:2000, Abcam, Cat. No.: 154385). After washing in TBS with Tween20®, sections were incubated for 30 minutes at room temperature with the appropriate HRP-conjugated secondary antibodies (donkey-anti-rabbit, Invitrogen by Thermo Fisher, Roskilde, Denmark, Cat. No.: 31458; donkey-anti-goat, Abcam, cat. No.: ab97110) and washed in TBS with Tween 20®, and counterstained with Meyers haematoxylin for 2 min. Visualization was achieved through peroxidase reaction using 3,3’-diaminobenzidine tetrahydrochloride (DAB substrate kit Abcam, Cat. No. ab64238).

### 2.8 Fluorescent immunohistochemistry

The procedure for immunofluorescence was carried out essentially as previously described, with few modifications (14). In brief, sections with small antral follicles were de-paraffinized in Neo-clear® (Sigma Aldrich, Soeborg, Denmark, cat. No.: 1.09843.5000)., rehydrated in graded ethanol (99%, 96% and 70%), followed by antigen retrieval for 20 min. in Tris-EGTA buffer in a microwave (10 mM Tris, 0.5 mM EGTA, pH 9). Nonspecific binding was inhibited with Dako Real Antibody Diluent blocking buffer for 30 min at room temperature (Agilent Technologies, Glostrup, Denmark, Cat. No.: S202230-2). Sections were incubated with primary antibodies overnight at 4°C in a humid chamber. Applied antibodies include CYP17A1 (1:200, Santa Cruz, cat.no.: SC-46084) and HSD3B2 (1:200, Abcam, Cat. No.: 154385). Sections were washed in TBS before incubation with secondary antibodies (donkey-anti-rabbit Alexa FluorTM 647, donkey-anti-goat Alexa FluorTM 568; Invitrogen by Thermo Fisher, Cat. No. A31573, A11057, 1:300) at room temperature for 45 min. in dark. After that, the sections were washed in TBS and then incubated with DAPI (1:5000, Invitrogen, Cat. No.: D1306) for 3 minutes in the dark. Sections were washed 3x3 min. in TBS in the dark and transferred to dH_2_O for 10 min in the dark before mounting. Antibody dilution buffers were used in place of primary antibodies as negative controls and showed no staining (**Supplementary Figure S2**). Images were captured on a fully motorized Olympus BX63 upright microscope with an Olympus DP72 color, 12.8-megapixel, 4.140x3.096-resolution camera and edited with the ImageJ software.

### 2.9 Statistical analysis

For statistical analysis of gene expression differences, microarray data and RNA-seq data were analysed using the limma software package (version 3.16) (21). The expression profiles of samples within various experimental groups were subjected to analysis through the moderated t-test, following linear model fitting. Genes with an adjusted *p*-value (*q*-value) below 0.05 and an absolute logarithmic fold change (log FC) greater than one were considered differentially expressed. All box plots in this study were created using the ggplot2 package (version 3.4.0) (22).

## 3 Results

### 3.1 Microarray - expression of steroidogenesis-related genes in different follicular stages

We conducted an analysis of microarray gene expression data encompassing a total of 68 samples, categorizing them according to the respective follicular developmental stages. Specifically, we focused on genes associated with steroidogenesis, and the summarized findings are presented in **Table 2**.

In early follicle development, we observed predominantly low expression levels of genes related to steroidogenesis. However, as the follicles progressed towards the preovulatory stage, there was a substantial increase in the expression levels of these genes, with notable exceptions being *HSD17B1* and *FSHR* (see **Figures 2A, B**). Notably, with the exception of *HSD17B1*, there were minimal significant differences in gene expression between mural granulosa cells and cumulus cells following ovulation induction, as evidenced by *q*-values exceeding 0.05 or log FC values less than 1 (see **Figure 2A**).

The expression profile of steroidogenic acute regulatory protein (*StAR*) exhibited a gradual rise in expression with increasing follicle size, culminating around the time of ovulation (see **Figures 2A, B**). This elevated expression was consistent in both mural granulosa cells and cumulus cells following ovulation induction, displaying statistical significance when compared to other time points (*q*-values > 0.05 or log FC < 1).

Furthermore, the expression pattern of cytochrome P450 family 11 subfamily A member 1 (*CYP11A1*) displayed an initial downregulation, followed by a sharp upregulation with a 48.5-fold increase in pre-ovulation induction follicles (*q* value < 0.001, log FC = 5.6). These heightened expression levels were maintained post-ovulation induction (see **Figures 2A, B**).

*CYP17A1* reached expression levels exceeding background levels exclusively in preovulatory follicles before ovulation induction, with all *q* values < 0.001 and log FC > 2. In contrast, in all other groups, the average *CYP17A1* expression was down to background levels (see **Figures 2A, B**).

The expression of cytochrome P450 family 19 subfamily A member 1 (*CYP19A1*) remained consistently low in both preantral and small antral follicles, with no statistically significant difference between them (*q* value > 0.05). However, a marked increase in *CYP19A1* expression was observed in preovulatory follicles compared to all other developmental stages, evident by all *q* values < 0.001 and log FC > 1.5 (see **Figures 2A, B**). Furthermore, after ovulation induction, the gene expression levels in both mural granulosa cells and cumulus cells surpassed those in the early stages of folliculogenesis, with significant statistical differences (all *q* values < 0.001 and log FC > 1) (see **Figures 2A, B**).

*HSD3B2* displayed low expression levels during the early stages of folliculogenesis but exhibited significant upregulation both pre-OI and post-OI (all *q* values < 0.001 and log FC > 2) (see **Figures 2A, B**). There was no statistically significant difference in expression between the pre-OI and post-OI periods (*q* values > 0.05 or log FC < 1) (see **Figure 2A**).

Hydroxysteroid 17-beta dehydrogenase 1 (*HSD17B1)* exhibited relatively high expression levels consistently throughout follicular development when compared to other genes (**Figure 2A**). The lowest average expression levels were observed in post-OI cumulus cells (**Figure 2B**), mirroring the levels seen in the preantral follicle group (*q* value = 0.008, log FC = 4.99) (**Figure 2A**). However, both of these were lower (all *q* values < 0.05 and absolute log FC > 1) than the levels observed in small antral follicles and preovulatory follicles, where no significant expression differences were evident (**Figure 2A**). Samples from the preantral follicle and mural granulosa cells (post-OI) group displayed substantial variability in *HSD17B1* expression (**Figure 2B**).

Expression of *AKR1C3* (*HSD17B5*) was close to or below the background level of noise in all follicle classes.

The expression patterns of *FSHR* and the *LHCGR* exhibited opposing trajectories. *FSHR* expression was significantly higher in preantral, small antral, and pre-OI follicles when compared to post-OI follicles, where expression was nearly absent (all *q* values < 0.001 and absolute log FC > 2) (**Figures 2A, B**). In contrast, *LHCGR* exhibited negligible expression during the early stages of folliculogenesis but peaked just before the induction of final follicular maturation (both *q* values < 0.001 and absolute log FC > 5) (**Figures 2A, B**). Subsequently, *LHCGR* expression decreased but remained at a high-level post-OI, albeit with some variability among samples (**Figures 2A, B**).

The expression of cytochrome b5 type A (*CYB5A*) was relatively highly expressed in preantral, small antral follicles and in follicles pre-OI. Notably, CYB5A expression significantly increased GCs as follicles advanced from the antral stage to the preovulatory stage. Furthermore, CYB5A expression in GCs collected at the time of oocyte aspiration displayed a significant increase compared to other follicle classes (**Figures 2A, B**).

The expression patterns of *POR*, *HMGCR*, and *LDLR* share a similar trajectory. These genes exhibited relatively low expression levels in preantral – and small antral follicles, but their expression significantly increased in GCs collected preovulatory follicles pre-OI. Subsequently, there was a slight reduction in their expression levels in mural and cumulus cells collected post-OI (**Figures 2A, B**).

Expression of *DHCR24* share the pattern of *POR*, *HMGCR*, and *LDLR* in preantral and small antral follicles with significant increase in follicles pre-OI, to a level that remained high in GCs and cumulus cells at oocyte pickup (**Figures 2A, B**).

### 3.2 RNA sequencing - expression of steroidogenesis-related genes in different follicular stages

**Figure 3:**
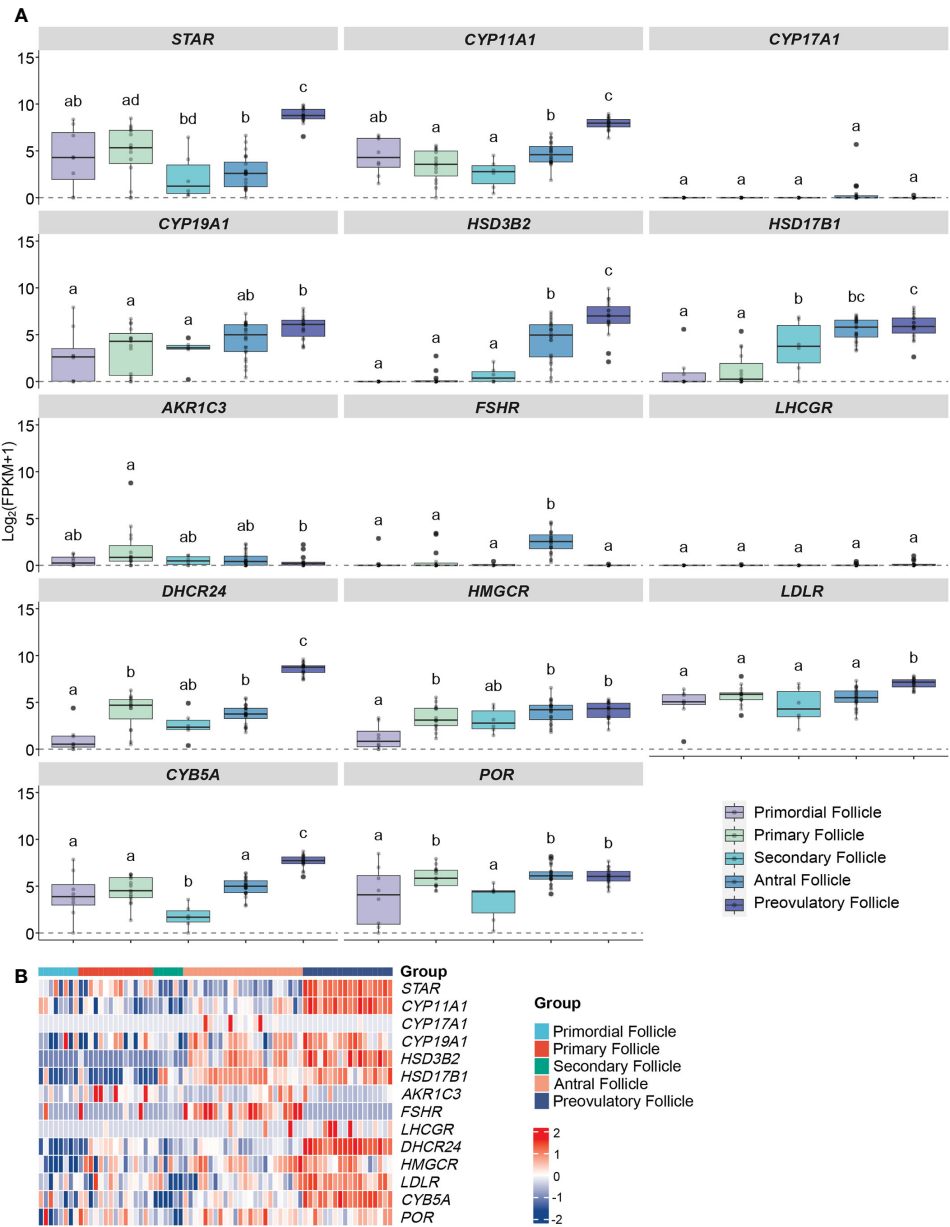
Expression profiles of steroidogenesis-related genes in human GCs at different follicular stages from RNA sequencing. **(A)** Boxplots of genes related to ovarian steroidogenesis in human follicles/GCs at different follicular stages. Log_2_ (FPKM+1) expression levels of each gene from the RNA-seq dataset are displayed. The x-axes show different groups of follicles, which are primordial follicles, primary follicles, secondary follicles, antral follicles and preovulatory follicles. Different letters between follicle/GCs groups indicate statistical significance. The background noise is shown as dot lines which is level 0. **(B)** Heatmap of genes expressed in human follicles/GCs at different follicular stage. The expression level of each gene between different samples is presented by the intensity of the colour. GCs, granulosa cells.

Data from a total of 71 samples were subjected to analysis, stratified into groups based on the stage of follicular development. The expression levels of genes associated with steroidogenesis are presented in **Table 3** and **Figure 3**. The expression patterns of the majority of steroidogenesis-related genes exhibited a progressive increase as follicles advanced in growth, ultimately peaking in preovulatory follicles just prior to ovulation induction (as illustrated in **Figures 3A, B**). Nevertheless, a noteworthy subset of genes displayed variability in their relative expression levels across samples collected from various stages of follicle development (as indicated in **Figure 3B**). It is important to note that in results presented here the groups of follicles are different from use used in microarray data. In both instances the background noise has been subtracted.

**Table 3:** RNA-seq analysis of expression level of selected genes in human follicle/granulosa cell at different stages.

log_2_ (FPKM+1)	Primordial Follicle	Primary Follicle	Secondary Follicle	Antral Follicle	Preovulatory Follicle
	(N=8)	(N=15)	(N=6)	(N=24)	(N=18)
*STAR*					
Mean (SD)	4.3 (3.3)	4.9 (2.8)	2.3 (2.5)	2.7 (1.7)	8.8 (0.8)
Median [Min, Max]	4.3 [0.0, 8.4]	5.3 [0.0, 8.5]	1.2 [0.2, 6.5]	2.6 [0.0, 6.6]	8.8 [6.5, 9.9]
*CYP11A1*					
Mean (SD)	4.4 (2.0)	3.4 (1.7)	2.5 (1.5)	4.7 (1.2)	7.9 (0.7)
Median [Min, Max]	4.3 [1.5, 6.7]	3.6 [0.0, 5.6]	2.8 [0.5, 4.5]	4.6 [1.9, 6.9]	8.0 [6.4, 9.0]
*CYP17A1*					
Mean (SD)	0.0 (0.0)	0.0 (0.0)	0.0 (0.0)	0.4 (1.2)	0 (0.1)
Median [Min, Max]	0.0 [0.0, 0.0]	0.0 [0.0, 0.0]	0.0 [0.0, 0.0]	0.0 [0.0, 5.7]	0.0 [0.0, 0.3]
*CYP19A1*					
Mean (SD)	2.7 (2.9)	3.5 (2.5)	3.2 (1.6)	4.5 (1.9)	5.8 (1.3)
Median [Min, Max]	2.6 [0.0, 7.9]	4.3 [0.0, 6.7]	3.6 [0.2, 4.7]	5.0 [0.4, 7.3]	6.1 [3.6, 7.8]
*HSD3B2*					
Mean (SD)	0.0 (0.0)	0.3 (0.7)	0.7 (0.9)	4.4 (2.2)	6.8 (2.0)
Median [Min, Max]	0.0 [0.0, 0.0]	0.0 [0.0, 2.7]	0.4 [0.0, 2.1]	5.0 [0.0, 7.4]	7.0 [2.0, 9.1]
*HSD17B1*					
Mean (SD)	1.0 (1.9)	1.2 (1.8)	3.8 (2.8)	5.6 (1.2)	5.9 (1.3)
Median [Min, Max]	0.0 [0.0, 5.6]	0.3 [0.0, 5.4]	3.8 [0.0, 6.9]	5.8 [3.3, 7.1]	5.9 [2.6, 7.9]
*AKR1C3*					
Mean (SD)	0.5 (0.6)	1.7 (2.3)	0.5 (0.5)	0.7 (0.7)	0.4 (0.6)
Median [Min, Max]	0.3 [0.0, 1.3]	0.9 [0.0, 8.8]	0.5 [0.0, 1.1]	0.4 [0.0, 2.3]	0.2 [0.0, 2.2]
*FSHR*					
Mean (SD)	0.4 (1.0)	0.6 (1.2)	0.1 (0.2)	2.5 (1.2)	0.0 (0.0)
Median [Min, Max]	0.0 [0.0, 2.9]	0.0 [0.0, 3.4]	0.0 [0.0, 0.4]	2.5 [0.3, 4.6]	0.0 [0.0, 0.1]
*LHCGR*					
Mean (SD)	0.0 (0.0)	0.0 (0.0)	0.0 (0.0)	0.0 (0.1)	0.2 (0.3)
Median [Min, Max]	0.0 [0.0, 0.0]	0.0 [0.0, 0.1]	0.0 [0.0, 0.0]	0.0 [0.0, 0.4]	0.0 [0.0.0, 1.0]
*DHCR24*					
Mean (SD)	1.1 (1.5)	4.1 (1.8)	2.6 (1.5)	3.7 (1.0)	8.5 (0.7)
Median [Min, Max]	0.5 [0.0, 4.4]	4.7 [0.5, 6.3]	2.4 [0.4, 4.9]	3.8 [1.8, 5.5]	8.8 [7.4, 9.6]
*HMGCR*					
Mean (SD)	1.2 (1.3)	3.4 (1.3)	3.05 (1.32)	4.00 (1.20)	4.10 (0.92)
Median [Min, Max]	0.8 [0.0, 3.3]	3.1 [1.2, 5.5]	2.8 [1.5, 4.8]	4.2 [1.8, 6.6]	4.3 [2.1, 5.3]
*LDLR*					
Mean (SD)	4.8 (1.7)	5.7 (1.0)	4.6 (1.9)	5.5 (1.0)	7.0 (0.6)
Median [Min, Max]	5.1 [0.8, 6.4]	5.9 [3.6, 7.8]	4.3 [2.1, 7.0]	5.5 [3.2, 7.3]	7.2 [6.1, 7.8]
*CYB5A*					
Mean (SD)	4.1 (2.5)	4.6 (1.4)	1.8 (1.2)	5.0 (1.0)	7.6 (0.7)
Median [Min, Max]	3.9 [0.0.0, 7.8]	4.5 [1.4, 6.3]	1.7 [0.0, 3.6]	5.0 [2.9, 6.4]	7.7 [6.0, 8.7]
*POR*					
Mean (SD)	3.9 (3.1)	6.0 (1.1)	3.4 (2.1)	6.2 (1.0)	6.0 (0.8)
Median [Min, Max]	4.1 [0.0, 8.5]	5.8 [4.5, 7.9]	4.4 [0.2, 5.4]	6.1 [4.2, 8.2]	6.0 [4.4, 7.7]

N, sample number; SD, standard deviation; Min, minimum value; Max, maximum value.

*StAR* expression was consistently observed in all five distinct follicular stages, with no statistically significant differences in expression levels between primordial, primary, and secondary follicles. However, a notable and significant increase in *StAR* expression was seen in preovulatory follicles when compared to the other stages (all *q* values < 0.001 and log FC > 3), as illustrated in **Figure 3A**. This trend aligns with the findings from our microarray data analysis of comparable follicle classes.

In contrast, the expression of *CYP11A1* did not exhibit significant variation during the early follicular stages, with *q* values exceeding 0.05 and log FC remaining below 1. Notably, its expression was at its lowest point in secondary follicles, followed by a rapid and substantial increase in preovulatory follicles, where it reached its highest expression levels (all *q* values < 0.001, log FC > 3), as depicted in **Figures 3A, B**. This pattern resembles the observations made in our microarray study for corresponding follicle classes.

The expression of *CYP19A1* did not significantly differ among the primordial, primary, secondary, and antral follicle stages, with *q* values exceeding 0.05 and log FC remaining below 1. Nevertheless, there was a slight increase in expression observed in preovulatory follicles, indicating a marginal advantage over the primordial, primary, and secondary stages (all *q* values < 0.05, log FC > 1), as shown in **Figure 3A**. This observation contrasts with our microarray data, which exhibited a more pronounced up-regulation of *CYP19A1* from small antral to pre-ovulatory follicles.

The *HSD3B2* expression exhibited a clear upward trend with follicle maturation, with significantly higher expression levels observed in antral and preovulatory follicles compared to the less mature stages (all *q* values < 0.001, log FC > 3, and *q* = 0.0003, log FC = -2.36, respectively), as illustrated in **Figures 3A, B**. This pattern closely mirrors the results obtained from our microarray data analysis.

Gene expression of *HSD17B1* showed a trajectory like that of the microarray data with no significant difference detected between antral- and pre-OI follicles (*q* value = 0.76, log FC = - 0.35) (**Figures 3A, B**).

*CYP17A1* and *LHCGR* remained silent throughout the entire follicular development while *FSHR* showed relatively high expression only in the antral follicle group (*q* values < 0.001, log FC > 2) (**Figure 3A**). The expression of *LHCGR* and *FSHR* differ from that of the microarray data.

Expression of *DHCR24* was most notably significantly increased from the antral stage to the pre-OI stage of follicular development (**Figures 3A, B**).

The expression patterns of *HMGCR* and *POR* shared a similar trajectory but in contrast to the microarray data levels were similar between the antral and pre-OI stage, while that of *LDLR* became significantly upregulated in pre-OI follicles in contrast to microarray data (**Figures 3A, B**).

The expression of *CYB5A* increased significantly from antral to pre-OI follicles with expression being present in preantral follicles showing a significant drop in secondary follicles (**Figures 3A, B**). These data resemble those of the microarray data for comparable follicle sizes.

### 3.3 Microarray - expression of steroidogenesis-related genes in GCs and cumulus cells after GnRHa or hCG administration

**Figure 4:**
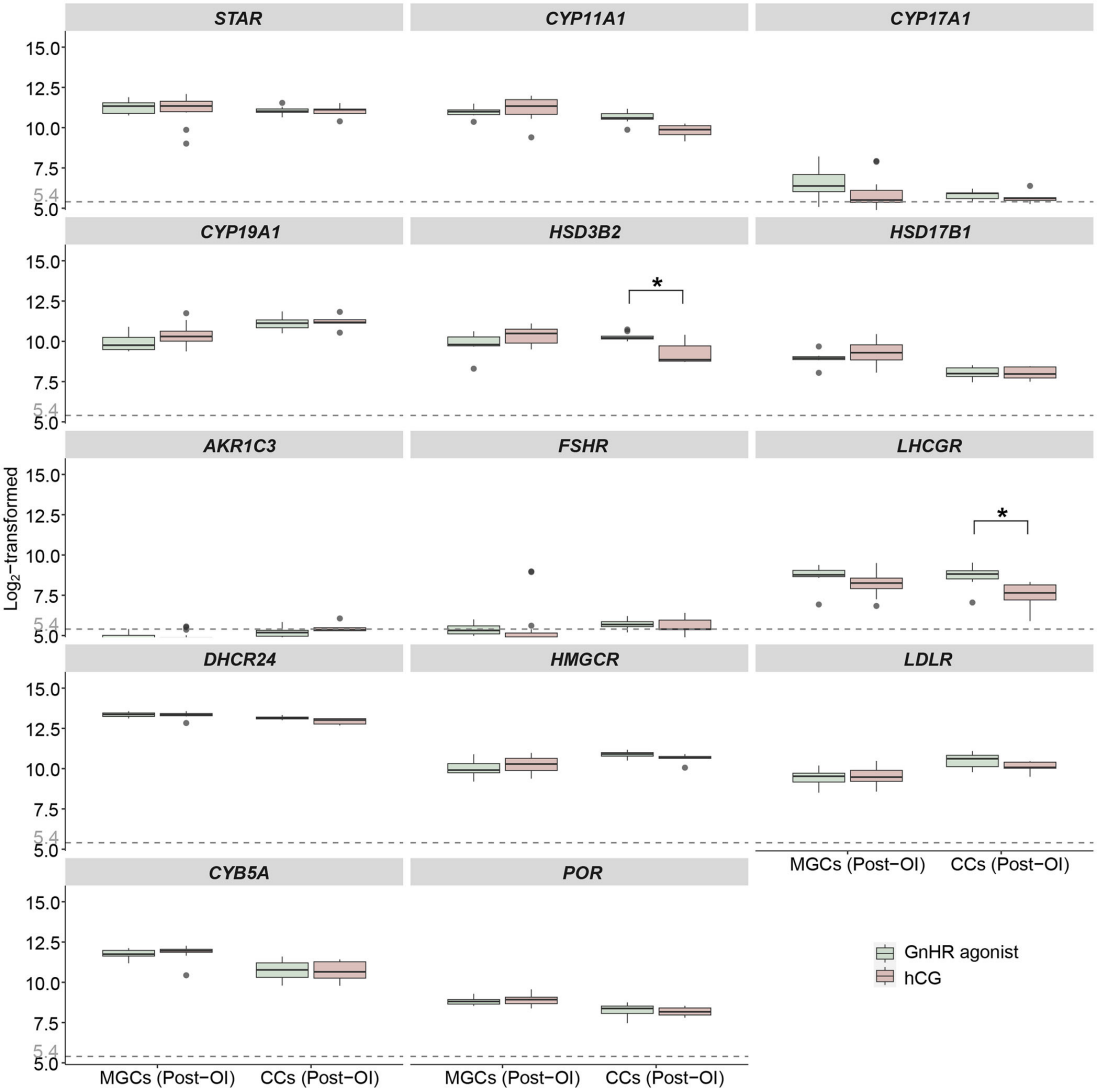
Expression profiles of steroidogenesis-related genes in mural GCs and cumulus cells after GnRHa or hCG induction from microarrays. Boxplots of genes related to ovarian steroidogenesis in mural GCs and cumulus cells collected at the time of oocyte aspiration. Log_2_-transformed expression levels of genes related to ovarian steroidogenesis from the microarray gene expression datasets are displayed. The x-axes show different samples of mural GCs and cumulus cells after ovulation induction. The green boxes represent sample data from patients who received GnRHa, while the pink boxes represent sample data from patients who received hCG for final maturation of follicles. Different letters between follicle/GCs groups indicate statistical significance. “*” indicates that two groups are statistically significant. Values below 5.4 are regarded as background noise. OI, ovulation induction; MGCs, mural granulosa cells; CCs, cumulus cells; GnRHa, gonadotropin-releasing hormone agonist; hCG, human chorionic gonadotropin.

The dataset comprised 39 samples, which were categorized and analysed based on cell type and the type of ovulation induction trigger (GnRHa or hCG). The influence of the ovulation trigger type on the expression of steroidogenesis-related genes was limited, as shown in **Figure 4** and **Table 4**. The only statistically significant differences observed between the two protocols were in the upregulation of *HSD3B2* and *LHCGR* genes in cumulus cells following GnRHa treatment (both *q* values < 0.001, absolute log fold change > 1) when compared to the hCG protocol.

**Table 4:** Microarray analysis of the expression levels of selected genes in human granulosa cells and cumulus cells after GnHR agonist or hCG trigger.

Log_2_-transformed	Mural granulosa cells (Post-OI)		Cumulus cells (Post-OI)	
	GnHR agonist	hCG	GnHR agonist	hCG
	(N=8)	(N=17)	(N=9)	(N=5)
*STAR*				
Mean (SD)	11.3 (0.4)	11.2 (0.8)	11.1 (0.3)	11.0 (0.4)
Median [Min, Max]	11.3 [10.8, 11.9]	11.3 [9.0, 12.1]	11.0 [10.6, 11.5]	11.1 [10.4, 11.5]
*CYP11A1*				
Mean (SD)	11.0 (0.3)	11.2 (0.7)	10.6 (0.4)	9.8 (0.4)
Median [Min, Max]	11.0 [10.4, 11.5]	11.3 [9.4, 12.0]	10.6 [9.9, 11.2]	9.9 [9.2, 10.3]
*CYP17A1*				
Mean (SD)	6.5 (1.0)	5.9 (0.9)	5.8 (0.3)	5.7 (0.4)
Median [Min, Max]	6.4 [5.1, 8.2]	5.5 [4.8, 7.9]	5.9 [5.4, 6.2]	5.6 [5.3, 6.4]
*CYP19A1*				
Mean (SD)	9.9 (0.5)	10.4 (0.6)	11.1 (0.4)	11.2 (0.5)
Median [Min, Max]	9.8 [9.4, 10.9]	10.3 [9.4, 11.7]	11.1 [10.5, 11.9]	11.2 [10.5, 11.8]
*HSD3B2*				
Mean (SD)	9.8 (0.7)	10.3 (0.5)	10.3 (0.2)	9.3 (0.7)
Median [Min, Max]	9.8 [8.3, 10.6]	10.5 [9.5, 11.1]	10.2 [10.0, 10.7]	8.9 [8.7, 10.4]
*HSD17B1*				
Mean (SD)	8.9 (0.5)	9.3 (0.7)	8.1 (0.3)	8.0 (0.4)
Median [Min, Max]	9.0 [8.0, 9.7]	9.3 [8.1, 10.4]	8.0 [7.5, 8.5]	8.0 [7.5, 8.4]
*AKR1C3*				
Mean (SD)	4.8 (0.3)	4.8 (0.4)	5.2 (0.4)	5.3 (0.6)
Median [Min, Max]	4.7 [4.4, 5.4]	4.6 [4.3, 5.6]	5.2 [4.5, 5.8]	5.3 [4.3, 6.1]
*FSHR*				
Mean (SD)	5.4 (0.4)	5.4 (1.4)	5.7 (0.3)	5.6 (0.6)
Median [Min, Max]	5.3 [5.0, 6.0]	4.9 [4.5, 9.0]	5.7 [5.2, 6.2]	5.4 [4.7, 6.4]
*LHCGR*				
Mean (SD)	8.7 (0.8)	8.3 (0.7)	8.7 (0.7)	7.4 (1.0)
Median [Min, Max]	8.8 [6.9, 9.4]	8.3 [6.8, 9.5]	8.8 [7.0, 9.5]	7.6 [5.9, 8.3]
*DHCR24*				
Mean (SD)	13.3 (0.1)	13.3 (0.2)	13.2 (0.1)	12.9 (0.2)
Median [Min, Max]	13.4 [13.1, 13.6]	13.4 [12.8, 13.6]	13.2 [13.0, 13.3]	13.0 [12.7, 13.1]
*HMGCR*				
Mean (SD)	10.0 (0.5)	10.2 (0.5)	10.9 (0.2)	10.6 (0.3)
Median [Min, Max]	9.9 [9.2, 10.9]	10.3 [9.4, 11.0]	10.9 [10.5, 11.2]	10.7 [10.1, 10.9]
*LDLR*				
Mean (SD)	9.4 (0.6)	9.5 (0.5)	10.5 (0.4)	10.1 (0.4)
Median [Min, Max]	9.5 [8.5, 10.2]	9.5 [8.6, 10.5]	10.6 [9.8, 11.1]	10.1 [9.5, 10.5]
*CYB5A*				
Mean (SD)	11.8 (0.3)	11.9 (0.4)	10.8 (0.6)	10.7 (0.7)
Median [Min, Max]	11.7 [11.2, 12.1]	12.0 [10.4, 12.3]	10.8 [9.8, 11.6]	10.7 [9.8, 11.4]
*POR*				
Mean (SD)	8.8 (0.2)	8.9 (0.4)	8.3 (0.4)	8.2 (0.3)
Median [Min, Max]	8.8 [8.5, 9.3]	8.9 [8.4, 9.6]	8.4 [7.5, 8.8]	8.2 [7.8, 8.5]

OI, ovulation induction; GnRH agonist, gonadotropin-releasing hormone agonist; hCG, human chorionic gonadotropin; N, sample number; SD, standard deviation; Min, minimum value; Max, maximum value.

### 3.4 Chromogenic immunohistochemistry analysis

**Figure 5:**
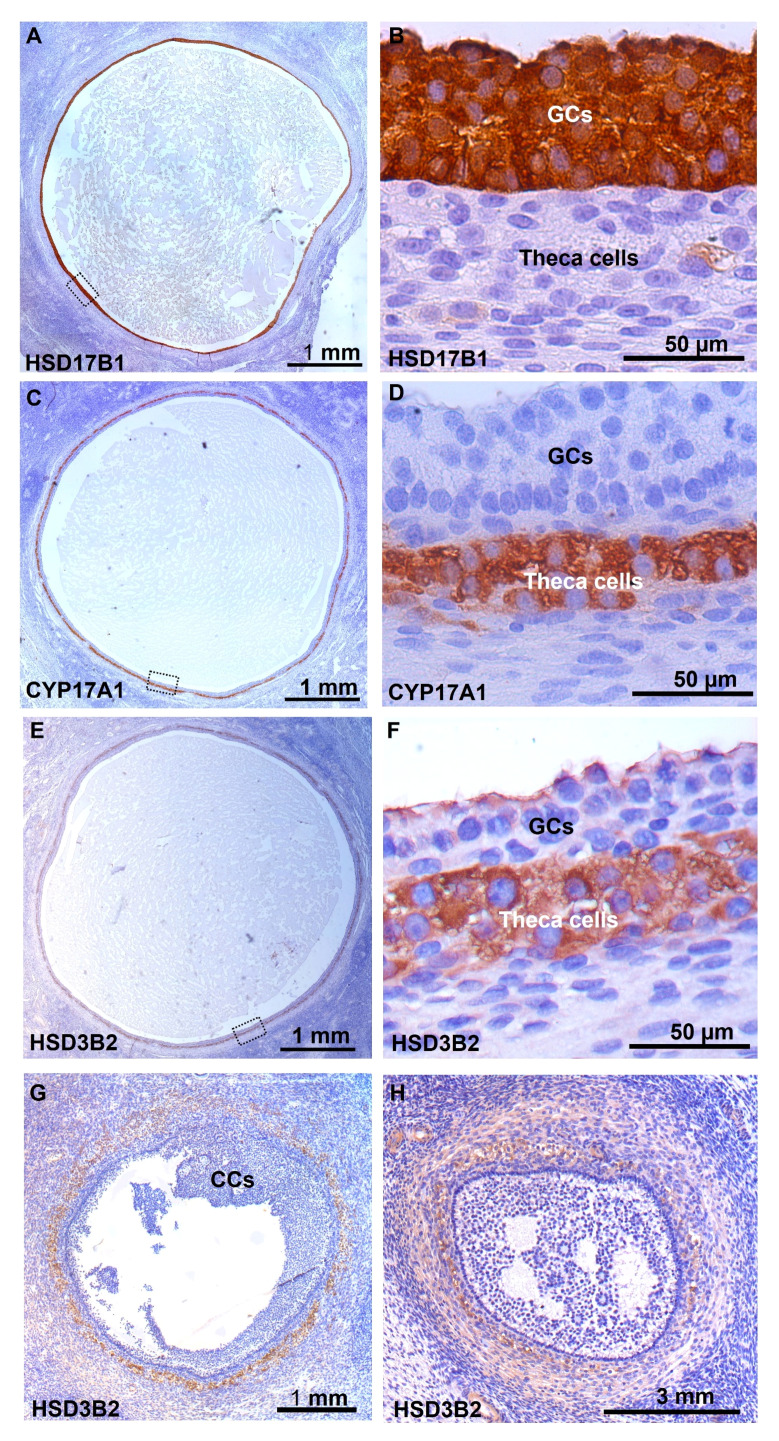
Immunohistochemical detection of enzymes involved in the steroidogenesis in human small antral follicles. **(A)** Detection of HSD17B1 specifically located to GCs in a 5 mm follicle, enlargement of the dotted box in **(B)**. **(C)** Detection of CYP17A1 specifically located to interna TCs, enlargement of the dotted box in **(D)**. **(E)** Detection of HSD3B2 in interna TCs, with a weak detection in GCs facing the lumen, enlargement of the dotted box in **(F)**. **(G)** Detection of HSD3B2 in externa TCs and stroma cells in a 1.5 mm follicle with no staining in GCs and cumulus. **(H)** In a 0.5 mm follicle HSD3B2 is detected in TCs and stroma cells surrounding the follicle. GCs, granulosa cells; TCs, theca cells.

Expression of HSD17B1 was exclusively detected in GCs from human small antral follicles (**Figures 5A, B**), CYP17A1 was exclusively detected in theca cells (**Figures 5C, D**). Expression of HSD3B2 was detected in the theca interna of follicles with a diameter of 5-6 mm, with weak staining observed in granulosa cells facing the lumen of the follicle (**Figures 5E, F**). In smaller follicles with a diameter of 1.5 mm, only theca externa and stroma cells expressed HSD3B2 (**Figure 5G**). The broader expression of HSD3B2 in theca and stroma cells surrounding the follicle was even more pronounced in small antral follicles with a diameter of 0.5 mm (**Figure 5H**).

### 3.5 Fluorescent immunohistochemistry analysis

**Figure 6:**
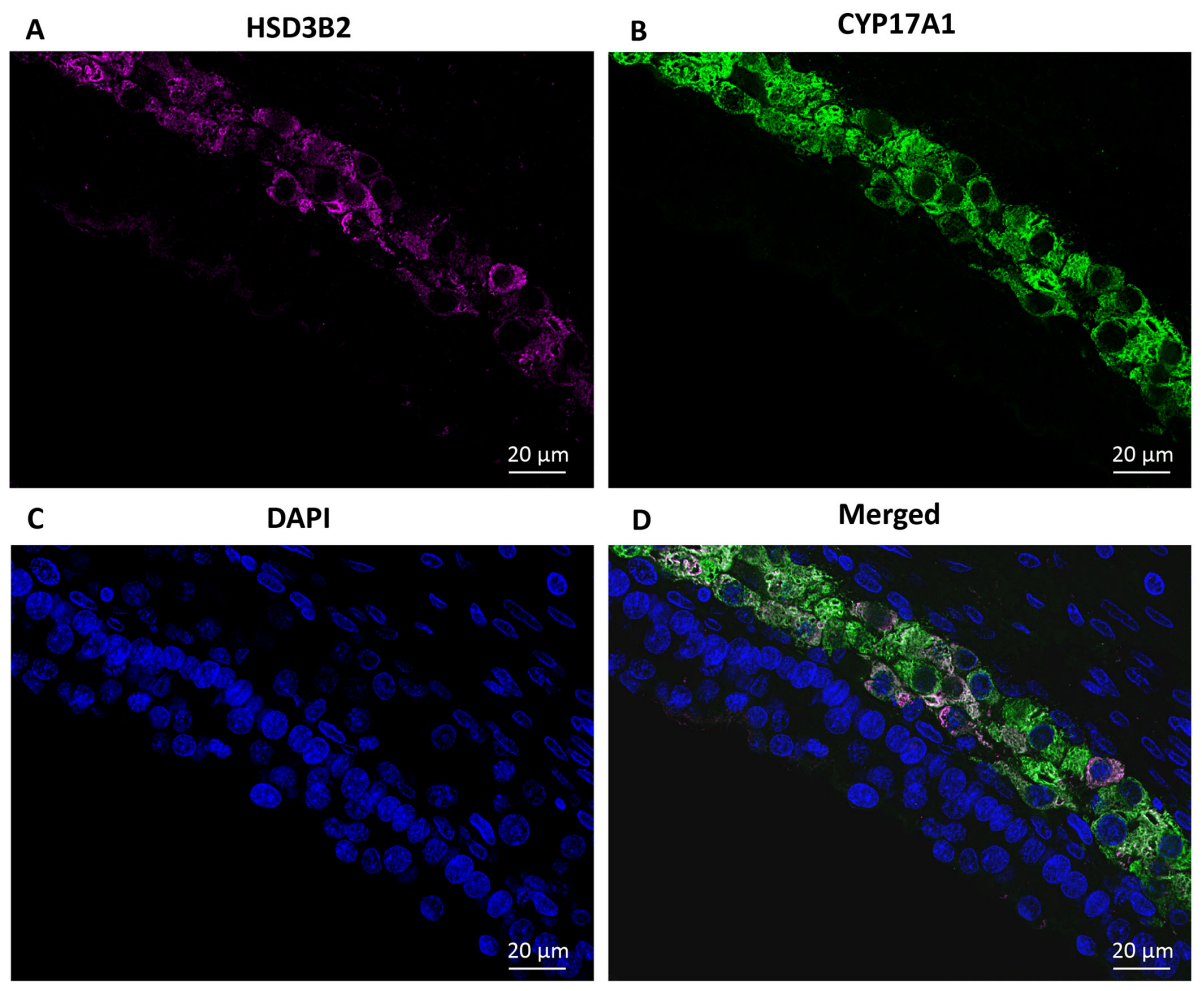
Immunofluorescence detection of HSD3B2 and CYP17A1 in a human small antral follicle. **(A)** Detection of HSD3B2 in TC interna in a 5 mm follicle (pseudocoloured magenta). **(B)** Detection of CYP17A1 specifically located to interna TCs (pseudocoloured green). **(C)** DAPI is used to label DNA (blue). **(D)** Merged image of **(A–C)** illustrating HSD3B2 and CYP17A1 co-localise specifically to a subset of TCs. No staining in other TC for both these two proteins. TC, theca cell.

The proteins HSD3B2 and CYP17A1 were detected exclusively in TCs in small antral follicles with a diameter of 5-6 mm (**Figures 6A, B**). DAPI was utilized to label DNA (**Figure 6C**) and was merged with images of HSD3B2 and CYP17A1 (**Figures 6A–C**), illustrating the co-localization of HSD3B2 and CYP17A1 to TCs (**Figure 6D**).

## 4 Discussion

This study reveals that the key enzyme responsible for P_4_ synthesis, HSD3B2, exhibits limited expression within GCs of preantral and small antral follicles. This expression pattern is supported by both microarray and RNA sequencing gene expression analyses, as well as immunohistochemical detection of HSD3B2 protein expression in human follicles. As these follicles progress towards the preovulatory stage, there is a notable increase in *HSD3B2* expression within GCs, coinciding with a rise in P_4_ levels within the follicle (4, 23, 24). This profile of P_4_ synthesis is enforced by the upregulation of *CYB5A*, which is significantly increased in both microarray and RNA-seq datasets as follicles transition from the antral stage to the preovulatory stage. Moreover, an augmented expression of *DHCR24* and *LDLR* in both types of datasets, along with *HMGCR* expression in the microarray data set, indicates increased substrate availability for ovarian steroidogenesis. These findings collectively support a substantial upregulation of P_4_ production in GCs as follicles progress from the antral to the preovulatory stage. As a corollary, human GCs contribute minimally to P_4_ production during the early follicular phase.

Immunohistochemical data reveals that HSD3B2 expression is predominantly observed in TCs surrounding follicles with diameters below 5-6 mm, with only minimal expression in GCs when follicles reach a diameter of 5-6 mm. This confirms and extends the notion that TCs exhibit greater steroidogenic activity than GCs during the initial half of the follicular phase, supported by a P_4_ to 17-OH-P_4_ ratio significantly below one (4).

Based on morphology, TCs were traditionally considered to consist of at least two distinct cell types: theca interna with spherical cells and theca externa with elongated cells. Recent studies have identified at least three different cell types in the theca cell layer (6). Whether 17-OH-P_4_ production depends on two different types of TCs, each expressing either HSD3B2 or CYP17A1, or whether HSD3B2 and CYP17A1 are co-expressed within one cell type in women remains to be determined. Nevertheless, immunofluorescence microscopy analysis in this study suggests that HSD3B2 and CYP17A1 localize to the same theca cells, implying that one type of TCs expresses both enzymes required for 17-OH-P_4_ production. This demonstrates that 17-OH-P_4_ production in human TCs occurs within a single cell and does not require cooperation between different cell types. Furthermore, we did not observe TCs, which did not simultaneously express both HSD3B2 and CYP17A1 suggesting that TC secretion of P_4_ is minimal.

Gene expression analyses of steroid-related genes, encompassing both microarray data and RNA-seq data, classified human follicles somewhat differently. In the RNA-seq study, antral follicles had a diameter of 0.2 to 10 mm, and preovulatory follicles exceeded 10 mm in diameter (19, 25, 26). In the microarray dataset, preantral follicles had a diameter of less than 0.2 mm, small antral follicles had a diameter of 5-10 mm, and preovulatory follicles, prior to ovulation induction, had a diameter of more than 17 mm.

While there are general similarities in expression patterns between the two data sets, there are a few exceptions. For instance, Zhang and colleagues did not observe *LHCGR* expression, possibly due to a lack of follicles from the late follicular phase in their dataset (19). These large follicles easily rupture during the isolation procedure and *LHCGR* only become upregulated in second half of the follicular phase (3). The absence of *FSHR* expression in preovulatory follicles in Zhang’s study contradicts previously published data (3) and cannot readily be explained.

These findings contribute to the ongoing discussion regarding the regulation of P_4_ during the follicular phase, particularly in the context of ovarian stimulation with exogenous gonadotropins and its importance in achieving pregnancy. Regardless of whether natural cycles (RNA dataset) or cycles involving ovarian stimulation (microarray dataset) are considered for antral and pre-OI follicles, similar results are obtained regarding the regulation of P_4_ production. Therefore, efforts to influence P_4_ synthesis during ovarian stimulation should focus on GCs, with production influenced by both FSH and LH activity.

The expression of genes related to ovarian steroidogenesis in GCs (*StAR, CYP11A1, HSD3B2, HSD17B1, CYP19A1*) combined with TC expression of *CYP17A1* in follicles reaching the antral stage suggests that the Δ5 pathway is active, and oestradiol synthesis may occur in preantral follicles. However, despite relatively high FSHR expression, oestradiol synthesis is kept at a low level until follicular selection at a diameter of around 8-10 mm (24). It has been hypothesized that the exceptionally high intrafollicular concentrations of AMH in follicles up to around 8-10 mm inhibit aromatase activity and oestradiol synthesis (27, 28).

Our findings validate and expand upon previous research conducted almost five decades ago, which indicated that the expression of HSD3B enzymes is predominantly confined to the TCs in human small antral follicles with diameters less than 10 mm (29, 30). In contrast, in preovulatory follicles with larger diameters ranging from 14 to 21 mm, the expression of HSD3B2 was observed within the GCs (29–31). In the context of preovulatory follicles, there have been reports suggesting that GCs and TCs exhibit similar levels of HSD3B2 expression (31), while another study proposed that HSD3B2 expression in GCs was reduced compared to TCs in preovulatory follicles (32).

When comparing GCs collected following two different methods of final follicular maturation, either through hCG or GnRHa triggers, our results indicate similar gene expression patterns for all enzymes involved in ovarian steroidogenesis. However, there are notable differences in the expression of *HSD3B2* and *LHCGR* within cumulus cells in the group that received hCG. Given that cumulus cells contribute only minimally to P_4_ production overall from the follicle, the functional significance of these expression differences remains challenging to ascertain but do suggest that gonadotropins are likely to also affect cumulus cell function.

It is also noticeable that expression of *STAR* and *CYP11A* in both datasets became significantly upregulated as follicles advanced from the antral to the preovulatory stage, showing that the capacity of the GCs to facilitate ovarian steroidogenesis is enhanced. The expression of these two genes is also present in preantral follicles and suggest that at least the initial steps in ovarian steroidogenesis are active in this stage of follicular development.

It is important to note that *CYP17A1* is expected to be absent in GCs and primarily localised to TCs. Data from both the microarray and the RNA results confirm that, but in pre-OI follicles, preceding the final maturation of follicles, the microarray data demonstrated gene expression above the background. Since our microarray data were validated by RT-PCR, which has been previously published (8–11), we hypothesize that this observation may be attributed to a minor contamination of TCs during the aspiration of preovulatory follicles, which had not yet been exposed to ovulation triggers, and the surrounding cells remained tightly packed.

It is a limitation that the present study lacks immunohistochemical analysis of large preovulatory follicles with a diameter exceeding 10-13 mm. However, we have been unable to successfully obtain such follicles for research purposes.

In summary, this study indicates that GCs exhibit limited progesterone synthesis during the early follicular phase, while certain TCs express both CYP17A1 and HSD3B2, contributing to 17-OH-P_4_ production. Around the follicular selection phase, approximately at a diameter of 8-10 mm, GCs begin to express *HSD3B2*, leading to an increase in P_4_ production supported by up regulation of *CYB5A* and genes regulation substrate availability for ovarian steroidogenesis. P_4_ synthesis by GCs intensifies with maturation, reaching high levels in follicles around the time of ovulation induction.

## Data availability statement

Publicly available datasets were analyzed in this study. This data can be found here: the microarray data (E-MEXP-3783, E-MTAB-2862, E-MTAB-2203, E-MTAB-1670) and RNA-seq data (GSE107746) are accessible in EMBL-EBI (https://www.ebi.ac.uk/) and GEO (https://www.ncbi.nlm.nih.gov/gds/), respectively.

## Ethics statement

The studies involving humans were approved by the Scientific Ethical Committee for the Capital Region (No. H-2-2011-044), the Danish Scientific Ethical Committee (SJ-156) and the Danish Ethical Committee (VN2004/61). The studies were conducted in accordance with the local legislation and institutional requirements. The participants provided their written informed consent to participate in this study.

## Author contributions

MZ: Conceptualization, Data curation, Formal Analysis, Investigation, Methodology, Resources, Software, Validation, Visualization, Writing – original draft. CA: Conceptualization, Funding acquisition, Methodology, Project administration, Supervision, Validation, Writing – original draft. FR: Formal Analysis, Investigation, Resources, Visualization, Writing – review & editing. JC: Writing – review & editing. SC: Writing – review & editing. LM: Conceptualization, Formal Analysis, Investigation, Methodology, Project administration, Resources, Software, Supervision, Validation, Visualization, Writing – original draft.
