# Supplementary material for: Expression of genes and enzymes involved in ovarian steroidogenesis in relation to human follicular development
Source: Front Endocrinol (Lausanne). 2023 Oct 26;14:1268248. doi: 10.3389/fendo.2023.1268248 (PMC10641382; doi:10.3389/fendo.2023.1268248)
Supplement: Supplementary file 1 [file DataSheet_1.docx]

Supplementary Material

Expression of genes and enzymes involved in ovarian steroidogenesis in relation to human follicular development

**Mengxue Zheng, Claus Yding Andersen*, Frida Roikjer Rasmussen, Jesús Cadenas, Søren Tvorup Christensen, Linn Salto Mamsen**

*** Correspondence:** Claus Yding Andersen: cya@yding.com

# Supplementary Figures and Tables

# Supplementary Tables

Supplement Table 1. Specific gene expression in human follicles/granulosa cells at different follicular stages from microarray analysis.

| **Log_2_-transformed** | **Preantral Follicle** | **Small Antral Follicle** | **Preovulatory Follicle (Pre-OI)** | **Mural Granulosa Cells (Post-OI)** | **Cumulus Cells (Post-OI)** |
| --- | --- | --- | --- | --- | --- |
|  | **(N= 17)** | **(N= 3)** | **(N= 9)** | **(N= 25)** | **(N= 14)** |
| ***PTPRC*** |  |  |  |  |  |
| Mean (SD) | 4.2 (0.2) | 4.6 (0.3) | 4.5 (0.6) | 4.6 (0.4) | 4.9 (0.4) |
| Median [Min, Max] | 4.2 [3.8, 4.5] | 4.6 [4.4, 5.0] | 4.2 [3.9, 5.7] | 4.7 [3.9, 5.2] | 4.8 [4.5, 6.2] |
| ***TSPY1*** |  |  |  |  |  |
| Mean (SD) | 4.5 (0.2) | 4.3 (0.3) | 4.5 (0.2) | 4.6 (0.2) | 4.8 (0.3) |
| Median [Min, Max] | 4.6 [4.2, 4.9] | 4.2 [4.0, 4.7] | 4.4 [4.2, 4.9] | 4.5 [4.3, 5.1] | 4.7 [4.5, 5.4] |
| ***SRY*** |  |  |  |  |  |
| Mean (SD) | 3.9 (0.4) | 3.91 (0.1) | 3.9 (0.3) | 4.4 (0.5) | 4.4 (0.5) |
| Median [Min, Max] | 3.9 [3.2, 4.5] | 4.0 [3.8, 4.0] | 3.9 [3.6, 4.4] | 4.4 [3.3, 5.3] | 4.5 [3.5, 5.4] |

OI, ovulation induction; N, sample number; SD, standard deviation; Min, minimum value; Max, maximum value.

Supplement Table 2. Specific gene expression in human follicles/granulosa cells at different follicular stages from RNA-Seq analysis.

| **log_2_ (FPKM+1)** | **Primordial Follicle** | **Primary Follicle** | **Secondary Follicle** | **Antral Follicle** | **Preovulatory Follicle** |
| --- | --- | --- | --- | --- | --- |
|  | **(N=8)** | **(N=15)** | **(N=6)** | **(N=24)** | **(N=18)** |
| ***PTPRC*** |  |  |  |  |  |
| Mean (SD) | 0.2 (0.1) | 0.1 (0.2) | 0.0 (0.0) | 0.0 (0.1) | 0.0 (0.0) |
| Median [Min, Max] | 0.0 [0.0, 0.2] | 0.0 [0.0, 0.6] | 0.0 [0.0, 0.0] | 0.0 [0.0, 0.5] | 0.0 [0.0, 0.1] |
| ***TSPY1*** |  |  |  |  |  |
| Mean (SD) | 0.3 (0.7) | 0.0 (0.0) | 0.0 (0.0) | 0.0 (0.1) | 0.0 (0.1) |
| Median [Min, Max] | 0.0 [0.0, 1.9] | 0.0 [0.0, 0.0] | 0.0 [0.0, 0.0] | 0.0 [0.0, 0.4] | 0.0 [0.0, 0.4] |
| ***SRY*** |  |  |  |  |  |
| Mean (SD) | 0.0 (0.0) | 0.0 (0.0) | 0.0 (0.0) | 0.0 (0.0) | 0.4 (0.4) |
| Median [Min, Max] | 0.0 [0.0, 0.0] | 0.0 [0.0, 0.0] | 0.0 [0.0, 0.0] | 0.0 [0.0, 0.0] | 0.4 [0.0, 1.4] |

N, sample number; SD, standard deviation; Min, minimum value; Max, maximum value.

#
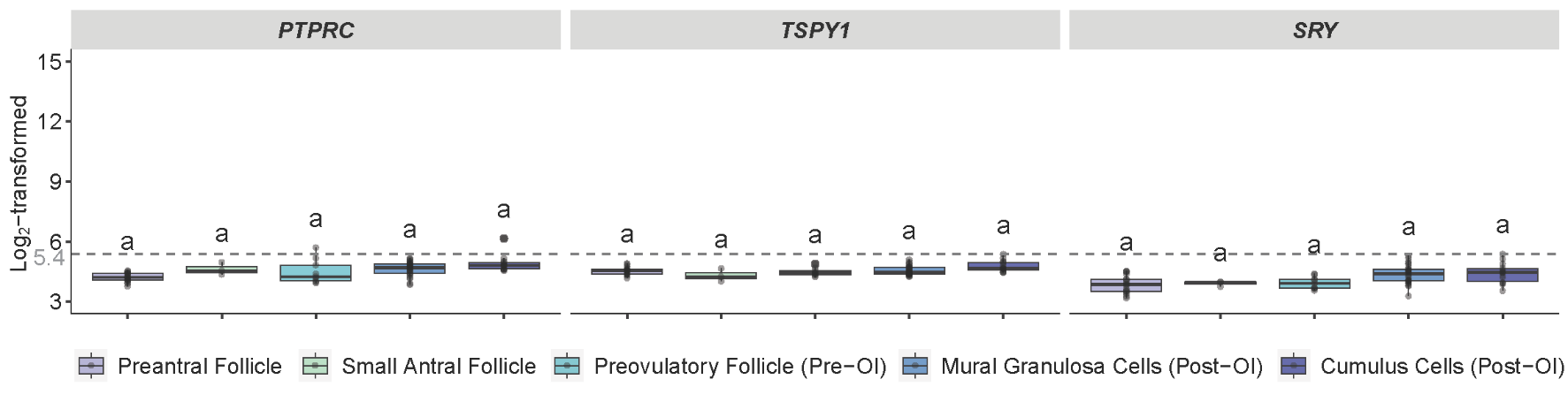
Supplementary Figures


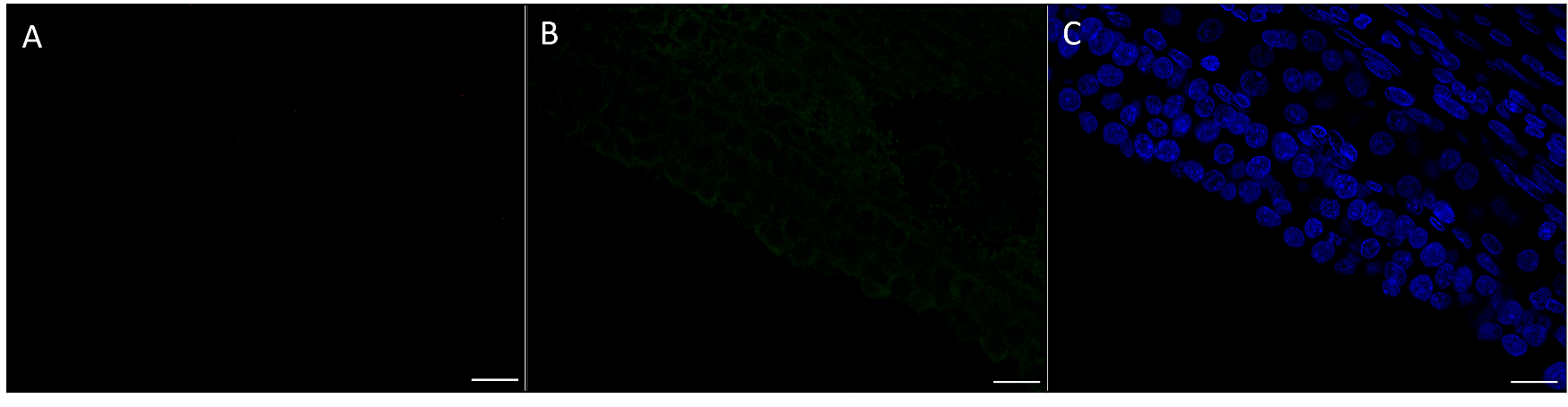
**Supplementary Figure 1. Specific gene expression in human follicles/GCs at different follicular stages from microarray analysis.** Log_2_-transformed expression levels of specific genes from the microarray gene expression datasets are displayed. *PTPRC* is the gene of the leukocyte-specific marker CD45, used to identify leukocyte contamination, with no statistical difference between samples. *TSPY1* and *SRY* are Y-chromosome-specific genes that were used to detect noise (background) in gene expression. There were no significant differences in the three genes among the five groups. GC, granulosa cell; OI, ovulation induction.

**Supplementary Figure 2. Representative negative controls immunofluorescence microscopy in the human small antral follicle. (A)** Donkey-anti-rabbit, wavelength 667nm (far-red). Pseudocoloured magenta. **(B)** Donkey-anti-goat, wavelength 568 nm (red). Pseudocoloured green. **(C)** Specific staining of DNA with DAPI in negative control. Scalebar 20 µm.


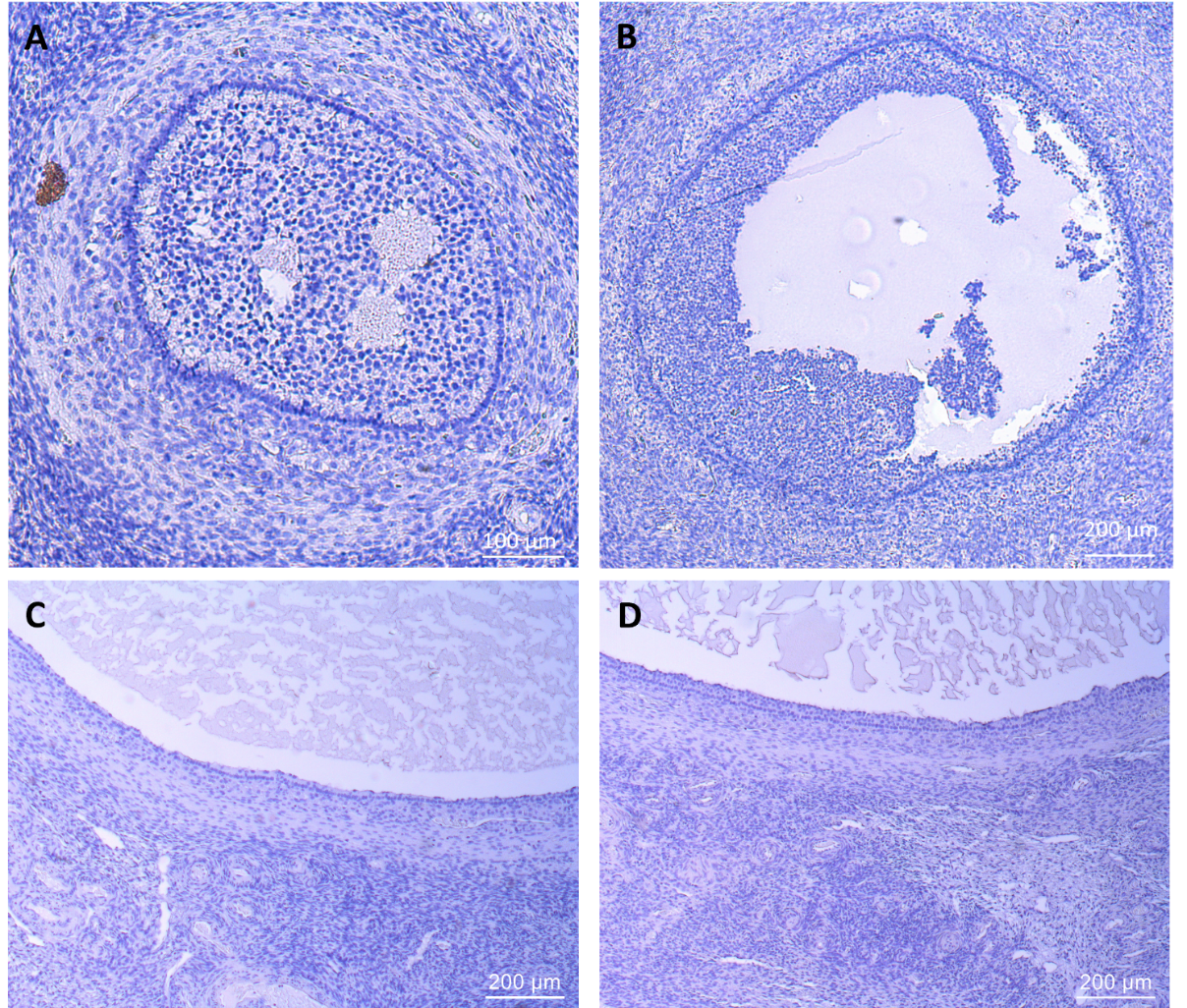
**Supplementary Figure 3. Negative controls IHC in human follicles.** The primary antibody was replaced with universal negative control serum (Biocare Medical, provided by Zytomed System GmbH, Berlin, Germany, Cat. No.: NC498H). **(A)** 0.5 mm. follicle, Donkey-anti-rabbit secondary antibody (Thermo Fisher, Roskilde, Denmark, Cat. No.: 31458). **(B)** 1.5 mm. follicle, Donkey-anti-rabbit secondary antibody (Thermo Fisher, Roskilde, Denmark, Cat. No.: 31458). **(C)** Donkey-anti-rabbit secondary antibody (Thermo Fisher, Roskilde, Denmark, Cat. No.: 31458). **(D)** Donkey-anti-goat secondary antibody (Abcam, Cat. No.: ab97110).
